# Tuning Electrostatic Gating of Semiconducting Carbon Nanotubes by Controlling Protein Orientation in Biosensing Devices

**DOI:** 10.1002/anie.202104044

**Published:** 2021-08-06

**Authors:** Xinzhao Xu, Benjamin J. Bowen, Rebecca E. A. Gwyther, Mark Freeley, Bella Grigorenko, Alexander V. Nemukhin, Johnas Eklöf‐Österberg, Kasper Moth‐Poulsen, D. Dafydd Jones, Matteo Palma

**Affiliations:** ^1^ Department of Chemistry and Materials Research Institute Queen Mary University of London London E1 4NS UK; ^2^ Molecular Biosciences Division School of Biosciences Sir Martin Evans Building Cardiff University Cardiff CF10 3AX UK; ^3^ Department of Chemistry Lomonosov Moscow State University Moscow 119991 Russian Federation; ^4^ Emanuel Institute of Biochemical Physics Russian Academy of Sciences Moscow 119991 Russian Federation; ^5^ Department of Chemistry and Chemical Engineering Chalmers University of Technology 41296 Gothenburg Sweden

**Keywords:** antimicrobial resistance, Biosensors, carbon nanotubes, protein engineering, protein orientation

## Abstract

The ability to detect proteins through gating conductance by their unique surface electrostatic signature holds great potential for improving biosensing sensitivity and precision. Two challenges are: (1) defining the electrostatic surface of the incoming ligand protein presented to the conductive surface; (2) bridging the Debye gap to generate a measurable response. Herein, we report the construction of nanoscale protein‐based sensing devices designed to present proteins in defined orientations; this allowed us to control the local electrostatic surface presented within the Debye length, and thus modulate the conductance gating effect upon binding incoming protein targets. Using a β‐lactamase binding protein (BLIP2) as the capture protein attached to carbon nanotube field effect transistors in different defined orientations. Device conductance had influence on binding TEM‐1, an important β‐lactamase involved in antimicrobial resistance (AMR). Conductance increased or decreased depending on TEM‐1 presenting either negative or positive local charge patches, demonstrating that local electrostatic properties, as opposed to protein net charge, act as the key driving force for electrostatic gating. This, in turn can, improve our ability to tune the gating of electrical biosensors toward optimized detection, including for AMR as outlined herein.

## Graphical abstract

Nanoscale protein‐based sensing devices designed to present proteins in defined orientations allowed the control of local electrostatic surface presented within the Debye length, and thus modulation of the conductance gating effect upon sensing protein targets.

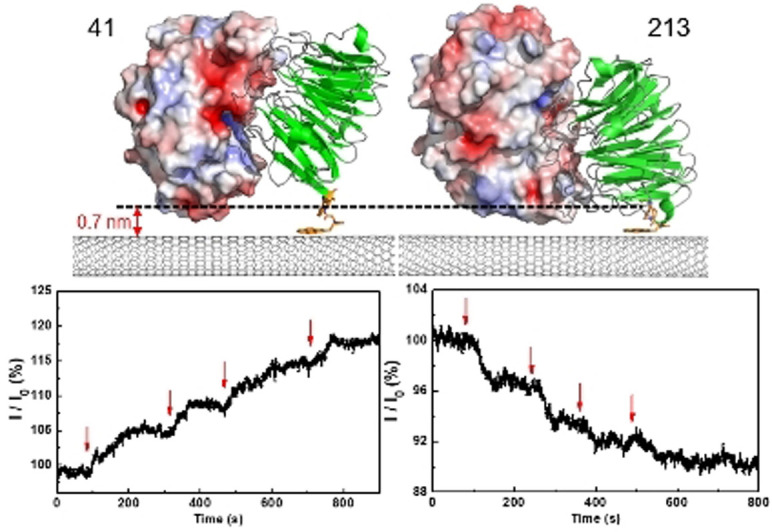

The construction of nanoscale field effect transistors (FETs) for sensing, whereby the gating voltage is replaced by a biomolecular event, offers huge potential for building high sensitivity, target‐specific, miniaturized, and label‐free biosensing devices.^[1]^ Protein surfaces are decorated with charged residues, whose distribution and area varies between proteins and within a protein, so generating a specific electrostatic signature. Therefore, there is great interest in developing systems that can sense and importantly differentiate these surfaces. Protein‐protein interactions are widespread in nature driving many important biological processes and form the basis for many diagnostic approaches. These highly specific interactions can in principle be used to electrostatically gate conductance far more effectively than they currently do by defining the interface between protein and the transducer, so forming the basis of greatly improved electrical‐based biosensors.[^1d^, ^1e^, ^2^]

While various semiconducting materials have been used in FET sensing devices, one‐dimensional (1D) materials offer advantages in terms of high surface area and restricted conduction pathways.[^1d^, ^1f^] Among 1D nanomaterials for sensing, single walled carbon nanotubes (SWCNTs) have emerged as excellent candidates, due to their high aspect ratios, restricted conductance pathways, appropriate size compatibility with biological analytes, the different strategies available for their functionalization, and the ease of integrating them into electronic circuits.[^1f^, ^2b^, ^3^] CNTs have been interfaced to different biomolecules,[^1d^, ^1f^, ^4^] with a particular focus on nucleic acids^[5]^ (e.g. DNA/aptamers) and proteins[^2a^, ^6^] (e.g. antibodies); this allowed biomolecular events to be transduced into measurable changes in CNT conductance.[^1d^, ^1f^, ^5a^, ^7^]

Despite previous work on FET biosensors, including CNT‐based ones, the approaches developed so far for the assembly of protein hybrids in device configurations typically suffer from the drawback of lacking control over protein interface orientation (Figure 1 a). This is particularly critical, as it does not allow us to fully take advantage of the unique surface distribution of electrostatic features of a protein, nor optimize communication between the protein(s) and the CNT. Furthermore, non‐specific attachment generates multiple orientations that can compromise sensing capability by, for example, sterically blocking access to a binding site, placing the incoming ligand beyond the Debye field length,^[8]^ or even destructively interfering by presenting differently charged surfaces. In this regard, the lack of geometric control is particularly important for nanoscale sensors where proteins constitute the sensing element and individual protein attachment variations can lead to major functional differences between devices. It is therefore of paramount importance to control the protein's site/residue that interfaces with the FET in order to define and understand the unique surface electrostatic signature driving gating upon sensing protein targets.

**Figure 1:**
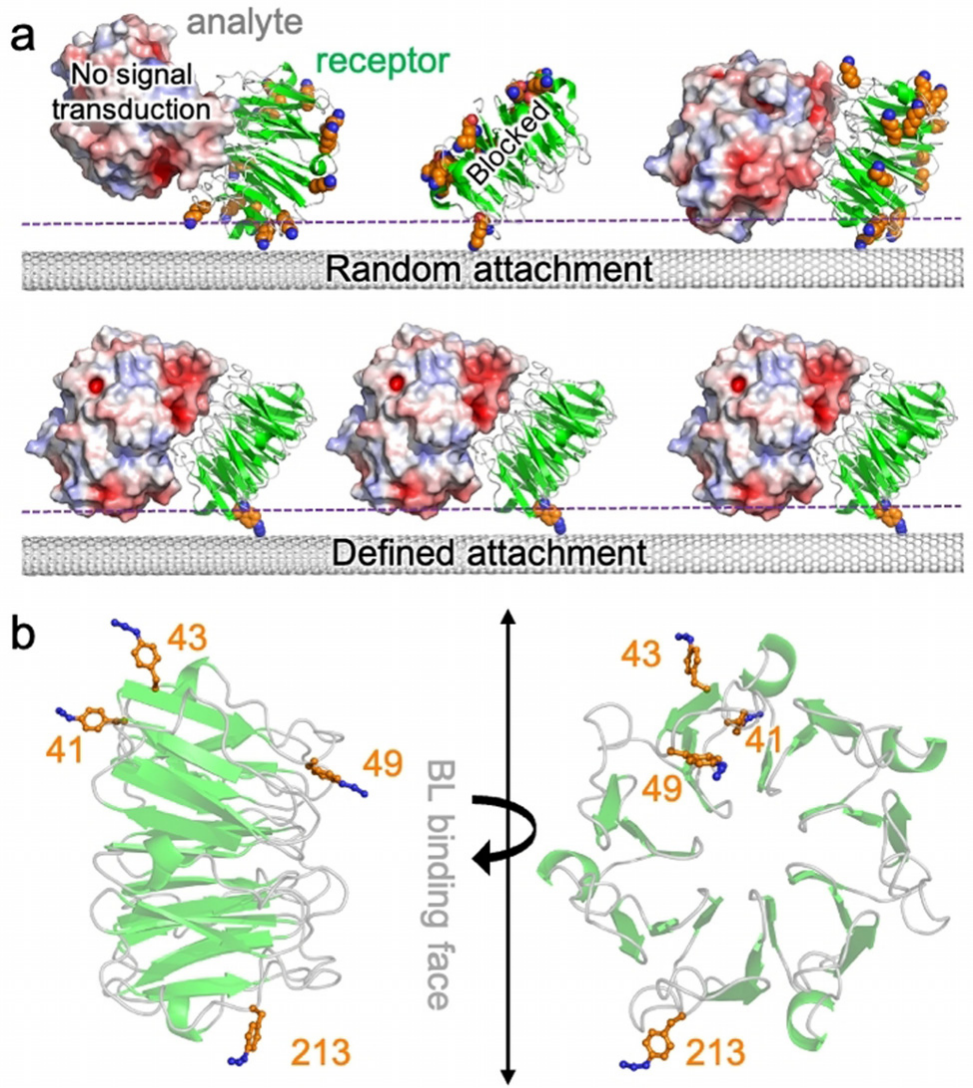
Design of BLIP2 (in green) interfacing sites. a) Different protein‐CNT interfacing approaches. Potential interface sites using standard amine attachment approaches (top); available lysine residues are shown as orange spheres. Shown are a binding orientation that does not result communication with CNT and another that blocks protein ligand binding (TEM‐1 shown in red, white and blue shades). Defined attachment using bioorthogonal ncAAs (bottom). The dashed purple line represents a putative Debye field length. b) Selected residues for replacement with AzF (see Supporting Scheme SI‐1 for details on incorporation) (PDB code 1jtd^[11d]^). The AzF models were built as described previously.^[6b]^

Previous work on attachment of proteins at defined residues demonstrated that CNT conductance can respond to local electrostatic changes close to the tube surface as a result of localized *intra*‐molecular changes, even down to the single‐molecule level.[^1c^, ^9^] However, these approaches were restricted to events close to the attachment residue of a protein directly attached to the SWCNT; for use in biosensing, *inter*‐molecular events such as protein‐protein interactions need to be monitored. This is a far more challenging proposition, as electrostatic events far from the attachment site need to be measured (Figure 1 a). Protein‐protein interaction surfaces areas are larger, and any steric blocking by the SWCNT will prevent protein ligand binding; those orientations that retain access to protein ligand binding site may result in the measured binding event being beyond the Debye field. This is particularly limiting in biological systems where high salt buffers may be needed so reducing Debye field distance. Thus, the initial choice of attachment residues becomes critical.

To address the challenge of monitoring inter‐molecular protein‐protein interactions through device response by sampling distinct electrostatic‐surface gating, we systematically tested how protein orientation dictates current response through a SWCNT‐FET device by defining the interface site on the capture protein. This is especially important given that most protein‐CNT interfacing approaches are essentially random, which leads to a heterogenous system comprised of non‐productive, non‐optimal and even mutually destructive orientations. Crucially, the device conductance increased or decreased depending on the selected designed orientation; this behavior is interpreted as due to changes of the local electrostatic surface presented within the Debye length upon binding,[^1c^, ^7b^, ^10^] and can support the identification of preferred proteins orientations for optimal sensing.

We have fabricated CNT‐protein FET biosensors with control over protein orientation in device configuration, focusing on the detection of a major cause of antimicrobial resistance (AMR), class A β‐lactamases (BLs). The BLs target and deactivate β‐lactam antibiotics (e.g. penicillin, ampicillin, amoxicillin), which are the mostly widely prescribed and utilized class of antibiotics. Thus, there is a real need to develop real time sensors for the presence of BLs in clinical samples, which will enable more appropriate and effective antibiotic at earlier stages of infection. We employed the BL inhibitory protein, BLIP2, that binds clinically prevalent class A BLs such TEM‐1 used here.^[11]^ By placing the non‐canonical amino acid (ncAA) p‐azido‐L‐phenylalanine (AzF)^[12]^ at four different designed positions in BLIP2 [see Scheme SI‐1 in the supporting information (SI)] we define the single‐site attachment of BLIP2 to the CNTs, in order to sample different electrostatic surfaces of an incoming BL (Figure 1 b). The benefit of using AzF is that multiple routes become available for tethering proteins to CNTs in a highly precise manner, including direct photo‐chemical attachment to the CNT sidewall^[6b]^ and click chemistry to a pyrene adduct.^[13]^ Here we use the click approach as unlike photochemical attachment, it does not introduce defects into the CNT; furthermore, our click chemistry approach has the advantage that any surface residue can be engineered to act as the interface site in one simple mutagenesis step, thiol‐based approaches may require further protein engineering to existing cysteine residues.[^1c^, ^9^]

Structural analysis of BLIP2 led to the selection of four residues being chosen to introduce AzF so as to investigate different facets of the protein‐protein interaction that gate SWCNTs conductance in response to the clinical prevalent BL, TEM‐1:^[14]^ Ala41, Ser43, Gly49 and Thr213 (Figure 1 b). Residues 41 lies on one side of the cone‐like BLIP2 structure, opposite to that of residue 213. Electrostatic surface modelling of BLIP2 complexed to the BL TEM‐1 demonstrates that by placing AzF at residues 41 or 213, very distinct surface charge profiles should be sampled (Figure 2). Gly49 lies at the BL binding interface so introduction of AzF and subsequent CNT binding should abolish any BL‐dependent conductance; this equates to a non‐productive interface configuration. Ser43 allows us to assess how even small changes to the attachment position can influence binding‐dependent conductance. BL enzyme inhibition assays revealed BLIP2^41AzF^ and BLIP2^213AzF^ retained near wild type, picomolar binding affinity, with BLIP2^43azF^ and BLIP2^49AzF^ having attenuated affinity in the low nanomolar range [see the SI for discussion, and Figure SI‐1].

**Figure 2:**
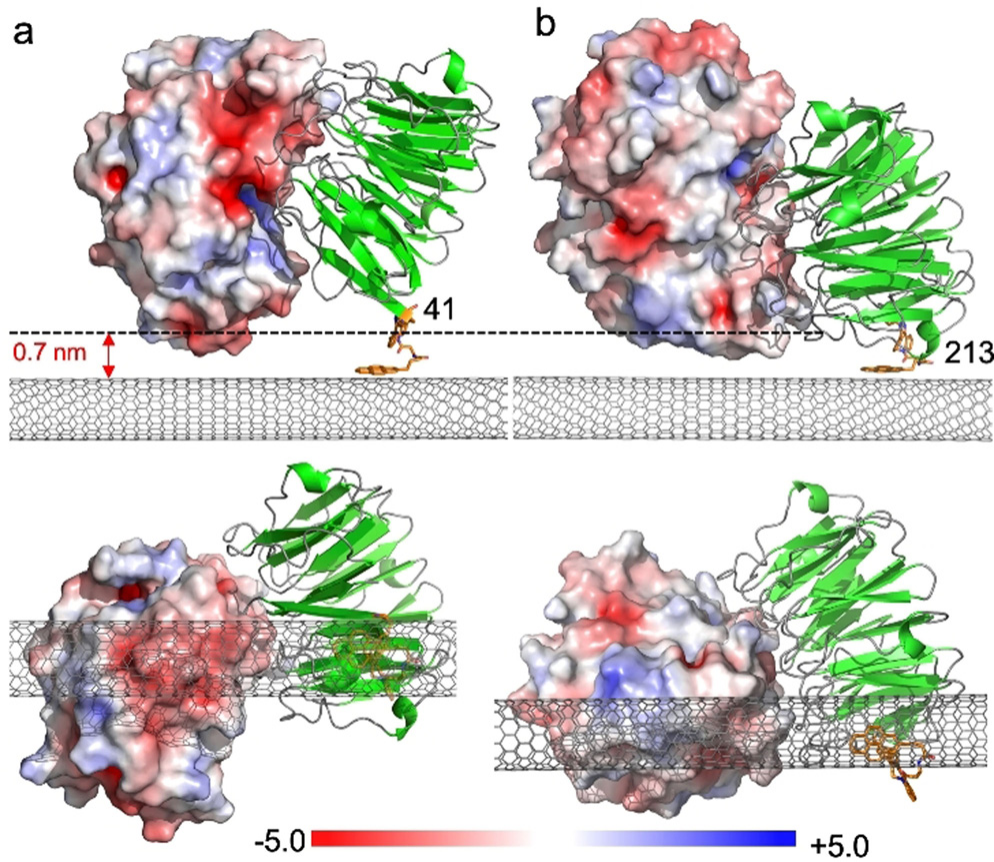
Models of TEM‐1 electrostatic surface presentation (shown in red, white and blue shades) on binding a) BLIP2^41AzF^ and b) BLIP2^213AzF^, both shown in green. A low energy configuration of the pyrene‐DBCO‐AzF moiety was docked on to the SWCNT surface followed by in silico ligation of the BLIP2 AzF variant into a sterically allowable conformation; the triazole conformation was compared to previously observed conformations found in crystals structures of AzF‐cyclooctynl linkages.^[15]^ The electrostatic surface (calculated using APBS electrostatic software^[16]^) of the TEM‐1 enzyme type is shown with a sliding scale of charge distribution. Top is a side‐on view of the complex with the Debye length shown as a dashed line. The pyrene adduct is coloured orange and the CNT grey. The lower panel shows the bottom up view of the complex with the CNT in the foreground.

CNT‐FETs were fabricated by casting solutions of sodium dodecyl sulfate (SDS)‐dispersed enriched semiconducting SWCNTs (sSWCNTs) on pre‐patterned electrodes pairs on doped silicon wafers, and immobilizing the nanotubes between 300 nm gap electrodes via dielectrophoresis (DEP): see the SI, and Figure SI‐2.^[5a]^ Typical transfer characteristics of the device are shown in Figure SI‐3, demonstrating that these sSWCNT‐based FETs are p‐type.

The pristine sSWCNTs where then coated in a 3:1 mixture of π‐stacking molecules pyrene‐butanol and dibenzylcyclooctyne (DBCO) modified pyrene‐amine.^[17]^ The pyrene butanol acts as a spacer, reducing BLIP2 density and minimizing non‐specific adsorption, while the DBCO allows attachment of BLIP2 via biocompatible strain‐promoted azide‐alkyne cycloaddition (SPAAC) (see Figure SI‐4 and Figure 3 a),[^13^, ^18^] without altering the electronic properties of the nanotubes by direct covalent attachment. Tethering of the BLIP2 variants to the FET devices, via the DBCO‐pyrene modified SWCNTs (Figure SI‐4) was monitored by atomic force microscopy (AFM). Figure 3 b shows a representative topographical profile of the device after BLIP2^41AzF^ attachment: the transverse height profile of the same tube sector increased by ≈3–4 nm (Figure 3 c), in line with the structure of BLIP2. Analysis of the other BLIP2 variants (Figure SI‐5) also showed nanotube transverse height increases of 3–6 nm upon protein attachment, again in line with what was expected based on the structure of BLIP2. AFM topographical analysis allowed us to estimate the average number of proteins attached to the nanotubes in each device to be ca. 40 to 80 proteins (see the SI and Figure SI‐6). No height increase was observed with the wild type protein (BLIP2^WT^) confirming the requirement of AzF for attachment (Figure SI‐7); moreover, no BLIP2‐AzF protein bound when nanotubes were coated with just pyrene‐butanol, hence ruling out off‐pathway attachment routes (Figure SI‐8). These results strongly suggest that our AzF‐containing BLIP2 variants attached to SWCNT‐FETs as designed.

**Figure 3:**
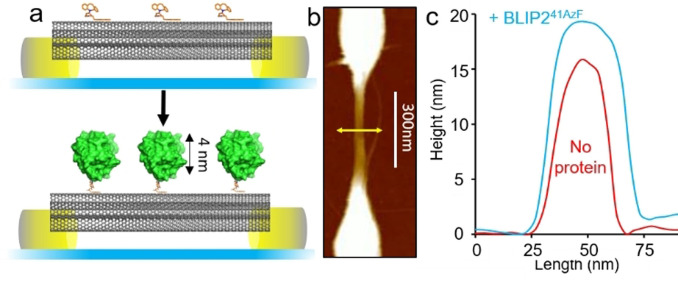
Attachment of BLIP2 to SWCNTs. BLIP2^41AzF^ is shown as a representative example with data for other BLIP2‐AzF variants in the Supporting Information (Figure SI‐5). a) Schematic of nanopatterned electrodes bridged by SWCNTs and attachment of BLIP2 variants to the sidewall of the nanotubes with defined orientation. The upper panel show nanotubes decorated with DBCO pyrene (orange) followed by attachment of BLIP2 (green). b) Representative AFM topographical image of the electrode with the yellow line indicating the axis across which height measurements were taken. c) AFM height profiles in device configuration before and after attachment of BLIP2^41AzF^. Additional height analysis of BLIP2^41AzF^‐SWCNT is presented in Figure SI‐5.

We then successfully demonstrated protein surface electrostatic gating of the p‐type SWCNT using our device configuration. Real‐time conductance measurements were performed for the four different attachment sites (and hence protein's orientations), monitoring current dependent changes upon addition of TEM‐1. Measurements were carried out in physiologically relevant, high ionic strength Dulbecco's phosphate buffered saline (DPBS) as a stringent test for our sensing configuration. The high ionic strength of DPBS restricts the Debye length (*λ*_D_) to circa 0.7 nm (see the SI),[^8b^, ^19^] which is smaller than the size of TEM‐1 (≈2×4 nm). This means only limited surfaces of TEM‐1 enter the screening layer of the device, influencing the conductance of the SWCNTs.^[10]^

Modelling of the BLIP2^41AzF^‐pyrene adducts docked onto the surface of the CNTs (see Figure 2 a and the SI) suggests that TEM‐1 binding will present a negatively charged acidic patch, comprised predominantly of residues E28, D35 and D38 within the Debye length. Figures 4 a and c show the real time, and concentration‐dependent, detection of TEM‐1 in DPBS buffer with devices functionalized with BLIP2^41AzF^. The current measured across the devices increased stepwise when TEM‐1 was added at increasing concentrations, which was mirrored when using serum doped with TEM‐1 (see the SI, Figure SI‐9). The change in conductance mirrored classical bimolecular ligand binding with largest relative binding signal observed at lower concentration additions. The first addition is equivalent to the detection of 100 moles of TEM‐1. Non‐functionalized CNT devices (i.e. without any BLIP2) did not induce any significant change in current on addition of buffer or TEM‐1, indicating that non‐specific adsorption was effectively suppressed in the CNT devices (Figure SI‐10); only devices functionalized with BLIP2s give an electrical response upon addition of TEM‐1.

**Figure 4:**
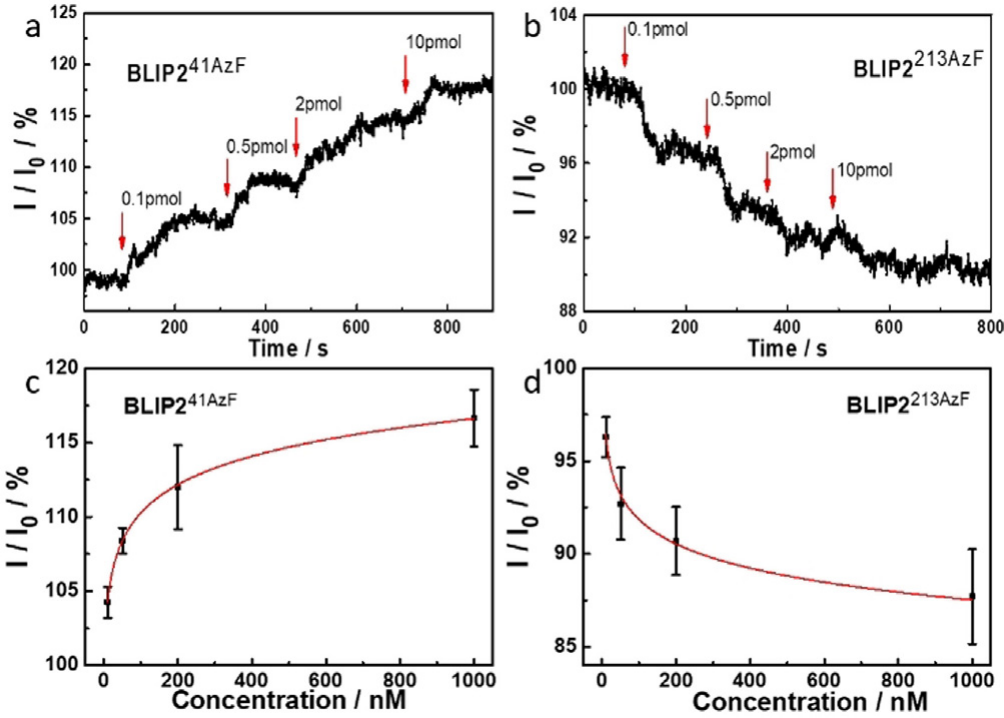
Orientation‐dependent gating of conductance. Conductance across SWCNT‐FETs functionalized with a) BLIP2^41AzF^and b) BLIP2^213AzF^. The 0.1, 0.5, 2, and 10 pmol amounts are equivalent to the following molar concentrations: 10, 50, 200 and 1000 nM respectively. The concentration dependent response for c) BLIP2^41AzF^and d) BLIP2^213AzF^. Addition of TEM‐1 at different concentrations is shown as vertical arrows on each conductance trace.

The observed conductance changes are in line with our molecular modelling of the protein‐CNT interface (Figure 2 a). The observed increase in current in our p‐type SWCNT‐FETs can indeed be rationalized as due to the higher negative charge density induced by TEM‐1 binding near the nanotube surface, which stabilizes a higher concentration of positive charges in the active channel, hence acting as an additional gating mechanism.[^1c^, ^7b^, ^17^, ^20^]

As shown in Figure 4 a and b, the current was not always reaching a stable plateau after the addition of TEM‐1. In future developments of our nanoscale sensing devices, engineering approaches could potentially minimize this via tuning the devices design and implementing a microfluidic system.

One major issue with random capture protein attachment is non‐productive orientations whereby the access to the binding site is blocked. We thus engineered the equivalent of a non‐productive binding interface in our devices. As shown in Figure SI‐11a, the BLIP2^49AzF^ configuration should block binding of TEM‐1 when tethered to the CNT. Indeed, devices functionalized with BLIP2^49AzF^ did not exhibit significant changes in current response upon addition of TEM‐1 at various concentrations (Figure SI‐11b).

The ability to optimize signal output through altering the capture protein interface residue and thus orientation is a distinct advantage to our approach compared to standard random attachment processes. To demonstrate how even small changes can potentially take the analyte beyond the Debye length hence affecting device performance, we shifted the AzF two residues along from 41 to 43. Using a similar modelling approach to BLIP2^41azF^, attachment of BLIP2^43azF^ results in a different binding configuration of TEM‐1 with respect to the SWCNT, with the acidic patch further from the tube surface, just at the boundary of the calculated Debye length (Figure SI‐12). This shift of just 2 residues from BLIP2^41AzF^ resulted in a relatively small signal change measured for BLIP2^43AzF^ on binding TEM‐1 (see Figure SI‐13), demonstrating the clear impact even minor changes in tethering site have on device sensitivity. While an upward trend in current is observed, lower signal response could be due to the incoming TEM‐1 binding being further from the nanotube surface as well as to the reduced affinity we observe in in‐solution (see Figure SI‐1) that in turn is either due to changes in protein affinity, obstruction of TEM‐1 binding site, or both.

Different protein electrostatic surfaces should elicit different conductance response. This was previously impossible to investigate using random capture protein attachment processes but is now feasible with our defined‐interfacing approach. BLIP2^41AzF^ presents a negatively charged acidic patch, that resulted in an increase in current in our p‐type SWCNT‐FETs (Figures 2 a and 4 a); by presenting a positive protein charge we should observe a decrease in current. In order to test this hypothesis, we functionalized our FETs with BLIP2^213AzF^ and assessed the device behavior to TEM‐1 binding. Modelling suggests that TEM‐1 presents a positively charged basic patch (Figure 2 b) comprised of residues R93, R94, H96 within the λ_D_ of BLIP2^213AzF^ functionalized SWCNTs. As shown in Figures 4 b and d, the current through these devices decreased stepwise as the concentration of the added TEM‐1 increased, that is, an opposite response if compared to devices functionalized with BLIP2^41AzF^. Due to the p‐type nature of our SWCNT‐FETs, the positive charge of the protein complex within the Debye length of the CNTs increases charge carrier repulsion decreasing transconductance.[^1c^, ^7b^]

The observed differences in the conductance behavior of the CNT‐FET devices presenting distinct protein's variants, can therefore be interpreted as due to changes in the local electrostatic surface accessible within the Debye length upon binding,[^1c^, ^7b^, ^8^, ^10^] and can support the identification of preferred proteins orientations toward optimized sensing. Lack of control over protein orientation can result in the simultaneous sampling of all the scenarios we have shown above: no response, minimal response, and even a cancelling effect due to a concomitant increase and decrease in current response. Furthermore, by sampling different protein orientations in distinct channels of the same device, future investigations can exploit the unique electrostatic surface signature of a protein to identify specific analytes in complex biological solutions. For example, with regards to BLs this can potentially allow the rapid identification of which BLs are present in a sample, and therefore inform on suitable antibiotic treatments; such information is not currently accessible by existing biochemical methods, even using ultrasensitive chemo‐luminescence approaches.^[20]^

In summary, we fabricated SWCNT‐FETs to investigate the influence of a protein's controlled orientation and electrostatic surface features in the electrical detection of an incoming ligand, namely the sensing of a β‐lactamase enzyme involved in AMR. Four distinct BLIP2 variants were engineered to contain bioorthogonal reaction handles at specific residues allowing proteins to be tethered to SWCNTs in defined orientations. Devices functionalized with different BLIP2 variants successfully detected the β‐lactamase TEM‐1 through changes in conductance, with device performance dependent on BLIP2 attachment site, and hence orientation. Presentation of different TEM‐1 electrostatic surfaces within the Debye length led either to increase or decrease in conductance; this allowed biosensing of less than 100 molecules through electrostatic surface profiling of protein‐protein interactions. The strategy presented here is of general applicability for the control and detection of protein‐protein interactions in nanoscale device configurations. Defined and homogenous attachment allows distinctive conductance profiles to be sampled based on the unique electrostatic features of individual proteins, and can support the identification of preferred proteins orientations for optimal sensing. By avoiding random/uncontrolled orientations we can minimize non‐productive and destructive interactions, and therefore consistently fabricate biosensors with defined response. With regards to AMR detection, this has significant potential as BLIP2 binds a range of BL enzymes, each with their own specific electrostatic profile; this may in turn open up new opportunities for the development of AMR‐related diagnostic devices that can be used to quickly detect the presence of resistance biomarkers and so more effectively utilize appropriate antibiotics to treat bacterial infections. The ability to quantify BL levels will also allow us to more accurately probe any link between enzyme levels and AMR, something which microbiological and genetic testing approaches cannot currently achieve.

## Conflict of interest

The authors declare no conflict of interest.

## Supporting information

As a service to our authors and readers, this journal provides supporting information supplied by the authors. Such materials are peer reviewed and may be re‐organized for online delivery, but are not copy‐edited or typeset. Technical support issues arising from supporting information (other than missing files) should be addressed to the authors.

Supporting Information
